# Mechanical testing and comparison of porcine tissue, silicones and 3D-printed materials for cardiovascular phantoms

**DOI:** 10.3389/fbioe.2023.1274673

**Published:** 2023-12-01

**Authors:** Joël Illi, Marc Ilic, Anselm Walter Stark, Cornelia Amstutz, Juergen Burger, Philippe Zysset, Andreas Haeberlin, Christoph Gräni

**Affiliations:** ^1^ Department of Cardiology, Inselspital, Bern University Hospital, University of Bern, Bern, Switzerland; ^2^ School of Biomedical and Precision Engineering, University of Bern, Bern, Switzerland; ^3^ ARTORG Center for Biomedical Engineering Research, University of Bern, Bern, Switzerland; ^4^ Translational Imaging Center, Sitem Center, University of Bern, Bern, Switzerland

**Keywords:** patient-specific phantoms, 3D-printing, additive manufacturing, cardiovascular tissue, biomechanical testing, tissue properties, cardiovascular phantoms, uniaxial tensile test

## Abstract

**Background:** Cardiovascular phantoms for patient education, pre-operative planning, surgical training, haemodynamic simulation, and device testing may help improve patient care. However, currently used materials may have different mechanical properties compared to biological tissue.

**Methods/Aim:** The aim of this study was to investigate the mechanical properties of 3D-printing and silicone materials in comparison to biological cardiovascular tissues. Uniaxial cyclic tension testing was performed using dumbbell samples from porcine tissue (aorta, pulmonary artery, right and left ventricle). Flexible testing materials included 15 silicone (mixtures) and three 3D-printing materials. The modulus of elasticity was calculated for different deformation ranges.

**Results:** The modulus of elasticity (0%–60%) for the aorta ranged from 0.16 to 0.18 N/mm^2^, for the pulmonary artery from 0.07 to 0.09 N/mm^2^, and for the right ventricle as well as the left ventricle short-axis from 0.1 to 0.16 N/mm^2^. For silicones the range of modulus of elasticity was 0.02–1.16 N/mm^2^, and for the 3D-printed materials from 0.85 to 1.02 N/mm^2^. The stress-strain curves of all tissues showed a non-linear behaviour in the cyclic tensile testing, with a distinct toe region, followed by exponential strain hardening behaviour towards the peak elongation. The vessel samples showed a more linear behaviour comparted to myocardial samples. The silicones and 3D printing materials exhibited near-linearity at higher strain ranges, with a decrease in stiffness following the initial deformation. All samples showed a deviation between the loading and unloading curves (hysteresis), and a reduction in peak force over the first few cycles (adaptation effect) at constant deformation.

**Conclusion:** The modulus of elasticity of silicone mixtures is more in agreement to porcine cardiovascular tissues than 3D-printed materials. All synthetic materials showed an almost linear behaviour in the mechanical testing compared to the non-linear behaviour of the biological tissues, probably due to fibre recruitment mechanism in the latter.

## 1 Introduction

Besides patient education, clinical education, training, pre-operative planning and hemodynamic testing, cardiovascular phantoms are also used for device testing, including simulation and its validation. Traditionally, they were mainly manufactured focusing on anatomical accuracy, and therefore mainly rigid materials have been used. Compliant phantoms became more available with the introduction and increasing availability of novel manufacturing technologies, e.g., additive manufacturing (AM). In parallel, advancements in medical imaging and segmentation have led to the accessibility of “patient-specific phantoms” that could depict anatomical features and also represent the patient’s unique pathophysiological behavior of the biological tissues. This has resulted in an increasing interest in creating more accurate 3D-printed patient-specific phantom (3DPSP) materials and silicon mixtures that mimic physiological tissue properties depending on the application (Bernhard et al., 2022). Compliance or elasticity is one of the most critical parameter for 3DPSP materials, which denotes the relationship between reversible deformations and an applied load (Emig et al., 2021). Incorporating compliance as a parameter in such phantoms is essential since it defines their behavior in static or dynamic physiological deformation. In order to align the compliance levels of a phantom with physiological values, it is necessary to have reference data on tissue mechanics. This process usually involves modifying the thickness of materials to achieve the desired structural properties, such as compliance or distensibility, with the elastic modulus being the fundamental material characteristic underlying this adjustment.

In the majority of cases, the adjustment of material thickness to attain the desired physiological compliance or distensibility is informed by existing literature data (Redheuil et al., 2010). Nonetheless, presently accessible compliant AM materials tend to exhibit greater elastic moduli than cardiovascular tissue, necessitating a reduced wall thickness to achieve the desired structural behavior (Jahren et al., 2017; Zimmermann et al., 2021; Di Micco et al., 2022). This also applies to indirect AM methods, specifically the technique of casting silicone into AM negatives of the anatomy (Bernhard et al., 2022; Illi et al., 2022), where the commonly utilized silicones for cardiovascular phantoms seem to exhibit excessive elastic moduli. At present, there is a lack of comparative data between various synthetic materials and cardiovascular samples. The information is dispersed across multiple papers in the current literature and due to variations in setup, geometry and protocol between these papers, a meaningful comparison cannot be done. The availability of such data would establish the basis for the development of materials that closely emulate biological tissues. This, in turn, would enable the creation of phantoms that more accurately represent a patients or specific pathological conditions.

The aim of the current study is to compare the mechanical properties of AM materials, silicones, and different silicone mixtures to allow for a novel approach to biological cardiovascular tissue mimic materials, whilst keeping the methodology (setup, geometry and protocol) as constant as possible for each material.

## 2 Materials and methods

### 2.1 Materials

#### 2.1.1 Porcine tissue

Whole porcine hearts with a median weight of 478.5 g (interquartile range (IQR) 447–526 g) from 14 pigs (same farm, cross breed of Swiss Edelschwein (∼Yorkshire) and Duroc, 6 month old, 110–115 kg) were obtained 1–2 h after euthanasia (in compliance with swiss animal rights and according to normal food processing regulations, by anaesthesia with electricity, followed by exsanguination) directly from the slaughterhouse and immediately cooled to 5°C. The hearts were further processed over a period of 1–3 days. Since the hearts were obtained as a by-product of the routine food industry, no ethical approval was needed.

#### 2.1.2 Silicones

In total, samples from 15 silicones and silicone mixtures were manufactured for testing. Besides commonly used silicones for cardiovascular phantom manufacturing, like Sylgard 184 (Dow Chemical Company, Midland, Michigan, United States) and Elastosil RT 601 (Wacker Chemie AG, Munich, Germany), Elastosil Vario 15 (Wacker Chemie AG, Munich, Germany), a range of mixtures, developed by us, as well as single compounds of Dragon Skin 30/20/10Slow and Ecoflex 00–30/20/10 (Smooth-On, Inc., Macungie, Pennsylvania, United States) were used in our testing series as outlined in Table 1. The selection of mixtures and single compounds of Smooth-On silicones was based on casting and mechanical testing trials, predating this investigation.

**TABLE 1:** Tested silicones and silicone mixtures Silicones and silicone mixtures used for testing, including the mixing ratios for the silicone mixtures, that have been avaluated in a pre-test study.

Single compound “pure” silicones		
Manufacturer	Compound	Shore hardnesses
Dow	Sylgard 184	A 48
Wacker	Elastosil RT 601	A 45
Wacker	Elastosil Vario 15	A 15
Smooth-On	Dragon Skin 30	A 30
Smooth-On	Dragon Skin 20	A 20
Smooth-On	Dragon Skin 10Slow	A 10
Smooth-On	Ecoflex 00–30	00–30
Smooth-On	Ecoflex 00–20	00–20
Smooth-On	Ecoflex 00–10	00–10

Developed two compound silicone mixtures		
Manufacturer	Compounds A and B	Ratio [%(Weight A)/%(Weight B)]
Smooth-On	Dragon Skin 30 + Ecoflex 00–10	50/50
Smooth-On	Dragon Skin 20 + Ecoflex 00–30	50/50
Smooth-On	Dragon Skin 10Slow + Ecoflex 00–30	80/20, 60/40, 40/60, 20/80

#### 2.1.3 Additive manufacturing materials

PolyJet printed Agilus30Clear (Stratasys Additive manufacturing company, Rehovot, Israel), currently the most commonly used direct AM material (Illi et al., 2022), was selected for testing, as well as Agilus30Black, to investigate the influence of colouring pigments, and IORA Model Flex 30A White (iSquared AG, Lengwil, Switzerland), the only free available competitor product for PolyJet printing. Those materials represent the softest AM materials currently freely available, with a Shore A value of 30. Furthermore, Polyjet is also currently the only freely available printing technology, which allows for flexible multi-material prints, which allows for simple incorporation of calcifications into the phantom.

As support material, Objet SUP706B Soluble Support (Stratasys Additive manufacturing company, Rehovot, Israel) was used for all prints.

### 2.2 Sample manufacturing

#### 2.2.1 Geometry

**FIGURE 1:**
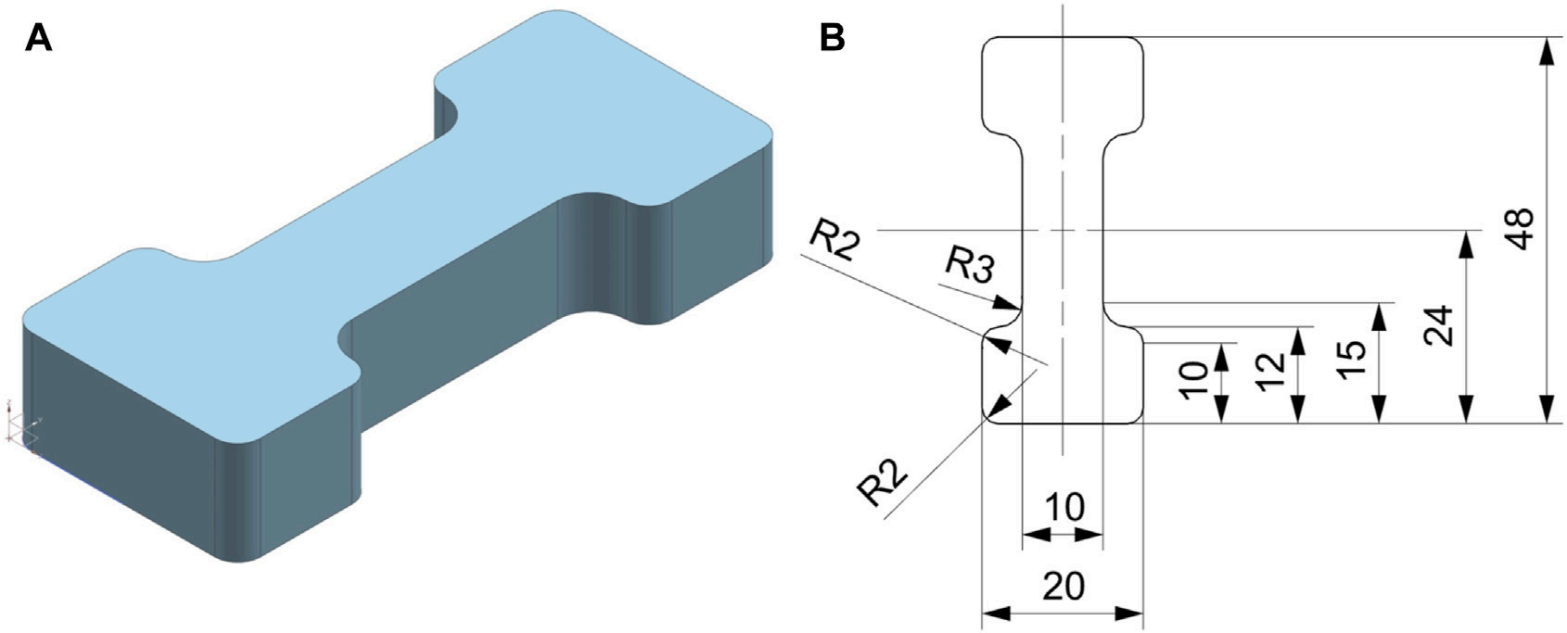
Designed dumbell sample geometry **(A)** Isometric view and **(B)** dimensioned drawing of the sample geometry. Dimensions are shown in mm, R = radius. Sample thickness was different for each material and, (in case of the biological tissue, different for each sample.

The sample geometry was a custom design based on normed tension testing geometries (aka. Dogbone, Dumbbell) (ASTM D638, 2021; DIN 53504 S1, 2017; ISO 37, 2017) and adapted to the anatomical (size) restrictions of the heart by shrinking the general dimensions and increasing the width-to-length ratio (Figure 1). The sample generation was also adapted per material to take into account the individual manufacturing process for cardiovascular phantoms. For example, the production method employed for the silicone samples differs from the guidelines in the norms mentioned above, as they were not generated through dye punching from a sheet. Instead, casting was used to ensure the applicability, as the phantoms are also casted. This change was done since the method of production can have an impact on the material properties. Those individual manufacturing techniques are elaborated in the following sections.

#### 2.2.2 Porcine tissue

**FIGURE 2:**
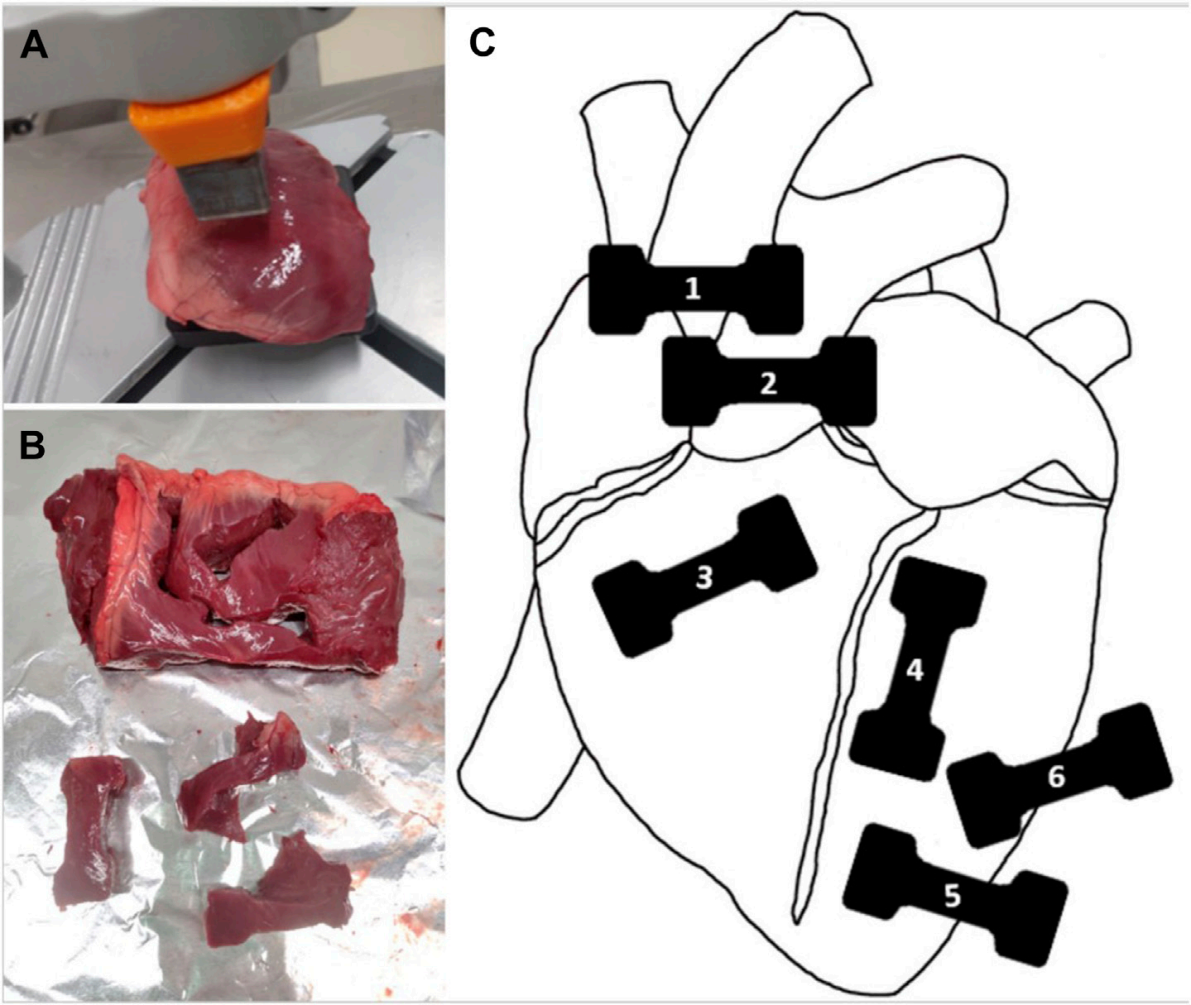
Porcine cardiovascular tissue sample generation and location **(A)** Dissected left ventricular wall in the die-cutter punch, **(B)** Left ventricular wall after punching, **(C)** Anterior view of the sample location (1 Aorta, 2 Pulmonary artery, 3 Right ventricle, 4 Left ventricle long-axis, 5 Left ventricle short-axis, 6 Left ventricle diagonally).

Porcine hearts were dissected into different tissues: aorta, pulmonary artery, right ventricle, and left ventricle (details are shown in Figure 2). The sample geometry was cut out using a custom-made blade punch and an AM die in a vertical drill press (Figure 2), for all of the four different dissected cardiovascular tissues. Of each heart, six samples were generated, including the aorta, pulmonary artery, right ventricle and three for the left ventricle. The samples for the left ventricles were extracted in different directions (long-axis, short axis, and diagonally), to account for fibre orientation. The last step of the sample preparation (only for the left ventricular samples of the heart 5–15) consisted of filleting the samples with an AM cutting guide into samples with 1 cm thickness to reduce in-sample thickness variation and ensuring comparability to other materials. Before testing, the sample thickness was measured without compression with a ruler at six locations (beginning, middle, and end of the gauge length on both sides), with an accuracy of ± 0.5 mm.

#### 2.2.3 Silicones

**FIGURE 3:**
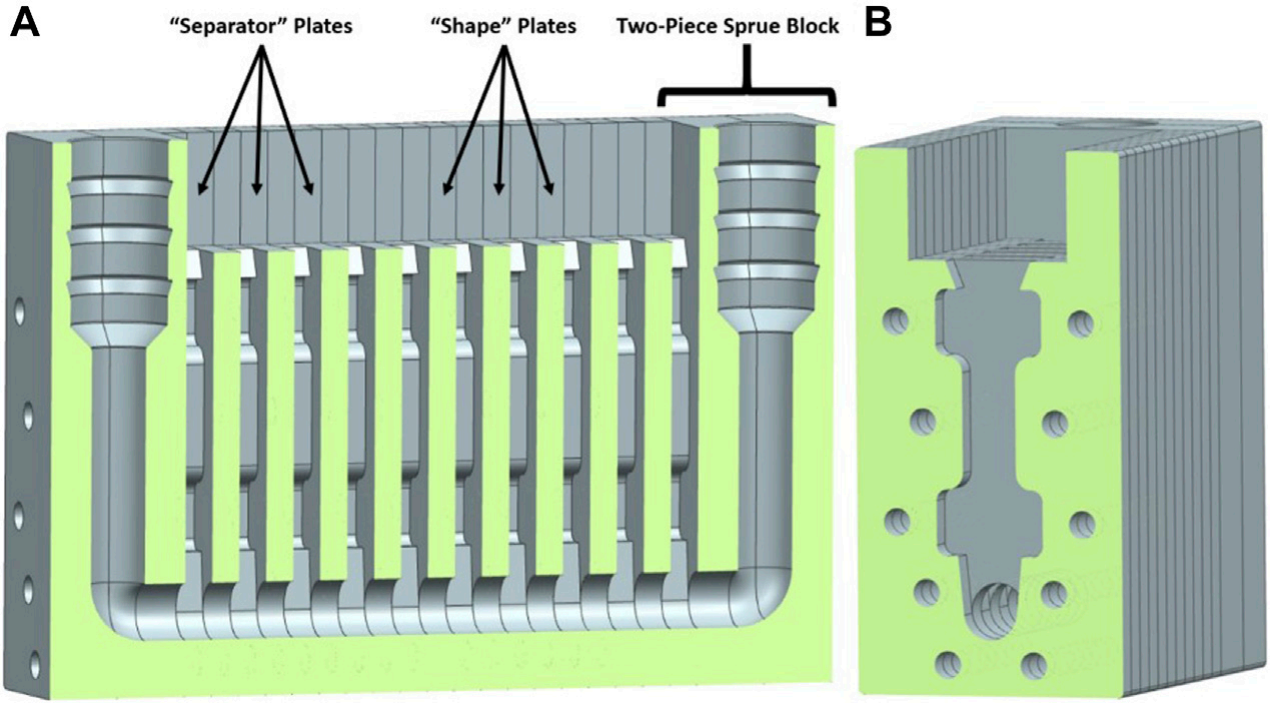
Gang mould for the manufacturing of the silicone samples **(A)** Longitudinal section through the gang mould assembly with 10 “shape” and 9 “separator” acrylic plates and the two 3D-printed two-piece sprue blocks at each end. **(B)** Cross-section of a single “shape” plate in the gang mould assembly.

For the silicone sample generation, a 10-piece gang-mould array (Figure 3) was manufactured, consisting of laser-cut acrylic “shape” plates and “separator” plates, as well as two 3D-printed two-piece sprue blocks. The mould was designed the ensure uniformity among the samples by filling all sample voids simultaneously from the bottom to the top. To further ensure repeatability and comparability of the casting process, a vacuum casting machine (mk technology System 1, MK Technology GmbH, Grafschaft, Germany) was used. After casting, all samples were post-cured according to the individual material specifications in the data sheet in a convection laboratory oven. The silicone samples were designed with a thickness of 5 mm, due to the limitation of the cutting depth of the laser of 5 mm for the moulds.

#### 2.2.4 Additive manufacturing materials

The dumbbell model for the AM samples was designed with a thickness of 9 mm. They were printed with a PolyJet printer (J750, Stratasys Additive manufacturing company, Rehovot, Israel) in three different orientations, with the print head travel direction along the tensile testing deformation direction, orthogonally and at a 45° angle. After printing, the samples were cleaned with a waterjet cleaning station, followed by a 4 h bath in a heated 2% sodium hydroxide alkaline cleaning solution with forced convection and a final clean with the waterjet.

### 2.3 Mechanical testing

**FIGURE 4:**
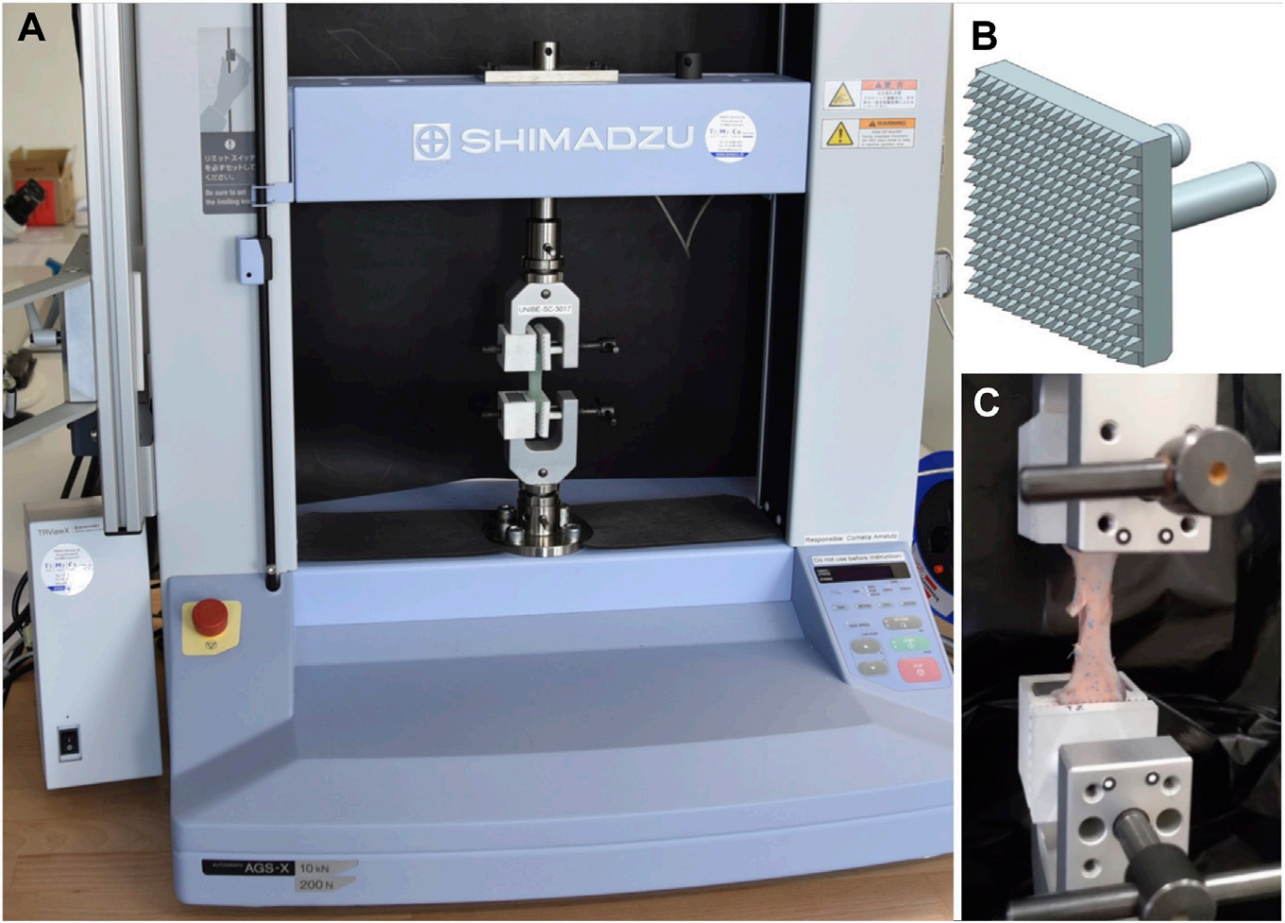
Uniaxial tension testing setup **(A)** Uniaxial tension testing setup with clamped Agilus30Clear sample. **(B)** Custom 3D-printed grips out of Polyamid for C-clamp vises with a pyramidal surface structure. **(C)** Detailed view of a vessel (i.e., aorta) sample during pre-testing.

For the material testing, a uniaxial tensile testing machine (Autograph AGS-X 200N, Shimadzu Corporation, Kyoto, Japan) with vise grips was used. The measurements were recorded with its proprietary software (Trapezium X Materials Testing Software v1.5.3, Shimadzu Corporation, Kyoto, Japan). Additionally to the standard vise grips, custom 35 mm × 32 mm 3D-printed Polyamid jaws with a 2 mm high pyramidal structure were used, to avoid slipping of the different materials (Figure 4). The clamping distance was set to 28 mm. The samples were first clamped in the upper vise in a centred position. Once the measured force had stabilized, it was reset to zero to eliminate any impact from the specimen’s weight. After the taring process, the 0-hold protocol was used to compensate for the compressive force on the load cell. This force is generated due to the sample elongation caused by the vise’s lateral compression during the lower vise’s tightening. Immediately after, the testing protocol was started. At the beginning of the protocol a slow preload of 0.05 N was done, followed by 10 cycles of loading (500 mm/min) to 60% deformation and unloading (250 mm/min) to 0% deformation. Those values were derived from the maximum travel speed of the uniaxial tensile testing machine and the limitation in relaxation speed of the artificial materials. This setup and protocol was applied to all measurements.

### 2.4 Data evaluation

After data was import into Matlab (The MathWorks, Inc., Massachusetts, United States), the force-displacement data was converted to stress-strain by dividing the force through to initial individual sample cross-sectional area, to obtain the nominal or engineering stress and in case of the deformation the initial clamping length of 28 mm was used, to get the nominal or engineering strain. As mentioned in Section 2.2.2 porcine tissue, the thicknesses of the porcine tissues were measured and calculated individually per sample, whilst with the artificial materials, we validated the designed thickness with one measurement on every sample, before testing, due to the much lower variability in thickness. To account for the adaptation effect (Lokshin and Lanir, 2009), which leads to overestimating the strength of soft elastic materials when cyclic testing, the first three cycles of the 10 repetitions were not used for analysis, as the first three cycles can be seen as preconditioning with stabilization during cycle 2 to 5 (Peña et al., 2009/04; Li et al., 2022; Dokos et al., 2002; Sommer et al., 2015). To account for the hysteresis and identify the individual cycles, each cycle’s loading and unloading portion was analysed separately by dividing the measurement data at the minimum and maximum strain into 10 loading and 10 unloading curves, respectively. The data from the obtained curves were linearly interpolated within the range of 0–0.6 strain. This was followed by averaging the interpolated data across cycles 4 to 10 for each sample and subsequently averaging them again across all samples for each tissue/material.

For the evaluation and comparison between the materials, the moduli of elasticity (slope of the stress-strain curve) were calculated for four regimes (0–0.2, 0.2 to 0.4, 0.4 to 0.6 and 0 to 0.6 strain).

## 3 Results

**FIGURE 5:**
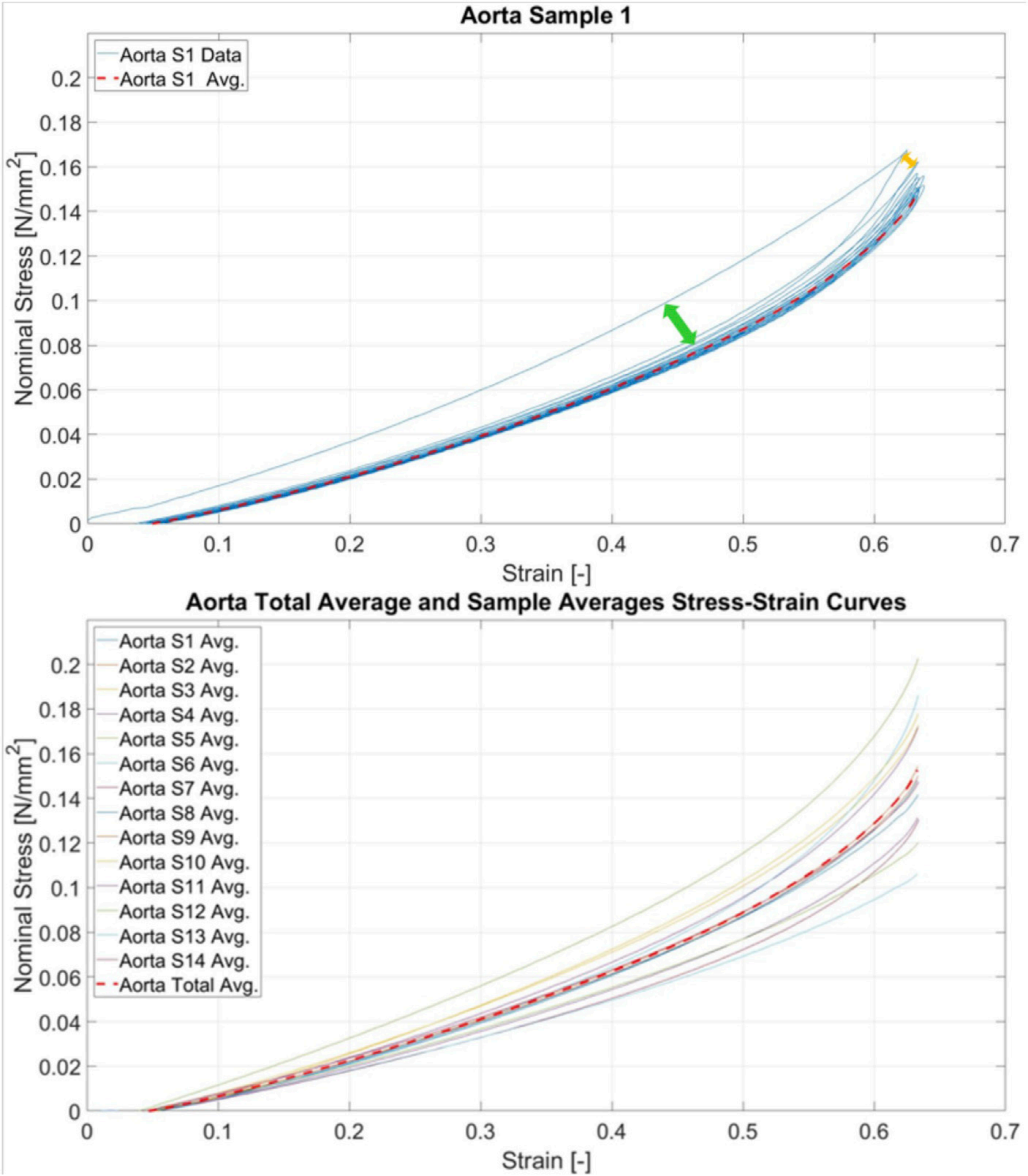
Testing cycle of the aorta sample 1 and averaged curves of all aorta samples Upper) Nominal stress-strain curve of the aortic sample 1 with dashed red line for the loading curve averaged over cycle 4 to 10. The green arrow displays the discrepancy between the loading and unloading curves (hysteresis) during the first cycle. The yellow arrow marks the decrease in stiffness between the first cycle and the second cycle, due to the adaptation effect. Lower) Averaged loading curves of all 14 aorta samples with the total average aorta loading curve over all samples in dashed red.

All samples individually showed differences in their loading and unloading curves, depending on the tissue or material to varying extents. During the first 2-3 cycles, the peak stress was always significantly lower than on the cycle before for the same strain. The tested materials showed a non-linear stress-strain behaviour, with some strain hardening depending on the material. This can be seen in the exemplary curve of the aorta samples in Figure 5.

### 3.1 Porcine tissue

Detailed information about the individual sample thicknesses as well as median and IQR can be found in Table 2.

**TABLE 2:** Thicknesses of the porcine cardiovascular tissue samples Median sample thickness by tissue and heart with IQR in brackets. The median and IQRs of the left ventricular samples were calculated separately for the first four hearts and the last ten hearts due to the change of procedure by filleting.

Heart	Aorta [mm]	Pulmonary artery [mm]	Right ventricle [mm]	Left ventricle long-axis [mm]	Left ventricle short-axis [mm]	Left ventricle diagonally [mm]
1	2.9	2.3	10.5	15.1	17.7	20.1
2	2.9	2.4	10.8	14.8	14.3	18.5
3	2.6	2.4	10.7	14.5	16.1	17.4
4	2.9	2.3	11.3	17.8	18.9	20.9
5	2.3	2.3	10.8	7.7	11.7	10.0
6	2.7	1.8	9.7	7.8	11.1	10.9
7	3.1	2.0	10.8	10.2	10.9	10.5
8	2.3	1.7	10.9	10.2	10.2	10.3
9	3.1	2.3	11.1	10.5	11.6	10.3
10	2.7	2.3	11.1	9.8	10.5	10.8
11	3.1	2.2	10.7	10.4	11.3	10.7
12	3.2	1.9	10.3	10.3	11.8	10.6
13	2.6	2.3	9.9	8.8	10.8	11.3
14	2.6	1.8	11.5	10.6	9.3	9.3
Median thickness native	2.8	2.3	10.8	15.0	16.9	19.3
	(2.6–3.0)	(1.9–2.3)	(10.5–11.0)	(14.8–15.8)	(15.6–18.0)	(18.2–20.3)
Thicknesses				10.2	11.00	10.5
filleted				(9.0–10.4)	(10.6–11.5)	(10.3–10.7)

The aorta samples had a median wall thickness of 2.79 mm. The measurements of these samples showed a strain hardening, non-linear material behaviour (Figure 5) with a short toe region in cas of the myocardial samples. The median elastic modulus ranged from 0.115 to 0.326 N/mm^2^ (Table 3).

**TABLE 3:** Elastic moduli of the porcine cardiovascular tissues Elastic moduli of the porcine cardiovascular tissues median, lower IQR and upper IQR over all samples for each tissue and for 4 different deformation ranges.

		L-IQR	Median	U-IQR
Tissue	Range	E-Modulus [N/mm^2^]		
Aorta	0%–20%	0.107	0.115	0.125
	20%–40%	0.181	0.198	0.214
	40%–60%	0.289	0.326	0.375
	0%–60%	0.190	0.213	0.243
Pulmonary Artery	0%–20%	0.021	0.026	0.029
	20%–40%	0.054	0.060	0.065
	40%–60%	0.111	0.124	0.135
	0%–60%	0.063	0.070	0.073
Right Ventricle	0%–20%	0.004	0.005	0.006
	20%–40%	0.026	0.029	0.034
	40%–60%	0.186	0.219	0.279
	0%–60%	0.073	0.083	0.105
Left Ventricle Long-Axis	0%–20%	0.094	0.128	0.184
	20%–40%	0.228	0.275	0.359
	40%–60%	0.281	0.281	0.281
	0%–60%	0.189	0.189	0.189
Left Ventricle Short-Axis	0%–20%	0.004	0.005	0.005
	20%–40%	0.027	0.030	0.034
	40%–60%	0.191	0.220	0.227
	0%–60%	0.074	0.086	0.089
Left Ventricle Diagonally	0%–20%	0.082	0.101	0.129
	20%–40%	0.141	0.179	0.261
	40%–60%	0.165	0.177	0.187
	0%–60%	0.103	0.121	0.131

**FIGURE 6:**
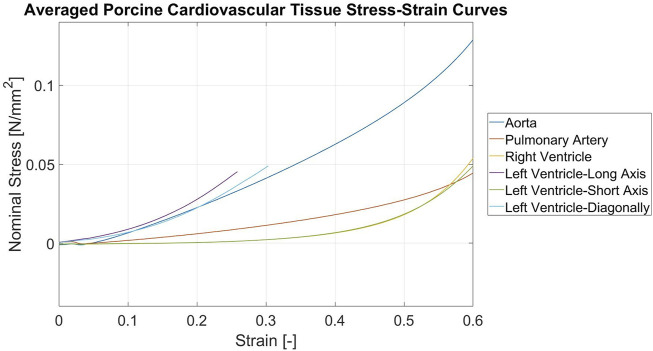
Stress-Strain curves of the porcine cardiovascular tissues Curves for the aorta, pulmonary artery, right ventricle, left ventricle long-axis, left ventricle short-axis, left ventricle-diagonally. All curves are averaged over 14 samples and the last 7 loading cycles.

The pulmonary artery samples had a median thickness of 2.33 mm. The behaviour was similar to the aorta samples, although at much lower stiffness, as shown by the graph in Figure 6 and the median moduli of elasticity, which range from 0.026 to 0.124 N/mm^2^.

The right ventricular samples had a median thickness of 10.75 mm. The difference in loading and unloading was similar to the vessels and only slightly more pronounced. The strain hardening behaviour was notably higher than for the vessel samples, with a longer toe region and a sharp increase in stiffness at the end of the measured deformation range, so it crossed the curve for the pulmonary artery tissue at 58% deformation. This can also be seen in the elastic modulus, which ranged from 0.005 to 0.219 N/mm^2^ for the different deformation ranges.

The short-axis samples were 16.88 mm before the resection and 11.00 mm for the following samples. The curve was almost congruent to the curve of the right ventricle, which is also shown by the range of the elastic moduli of 0.005–0.220 N/mm^2^ and only deviates slightly at the very end of the measured range.

The left ventricular long-axis samples had a median thickness of 14.96 mm before filleting and 10.17 mm afterward. Before the reduction, the diagonal samples had a median thickness of 19.29 mm and afterwards 10.54 mm.

The long-axis and diagonal samples all ruptured between 30% and 60% strain during the first cycle, most of them between 30% and 40% strain. Thus, the averaged curve was only calculated until 25% when the first samples started to rupture and by using data from the first loading cycle. Until then, the long-axis and diagonal samples showed similar behaviour. The curvature was similar to the aorta curve, although implying a much stiffer behaviour until the ruptures.

For the moduli, the last value before the rupture was taken. Thus, there was only one sample per orientation for which the 40% to 60% and 0%–60% strain modulus of elasticity could be calculated. It ranged from 0.128 to 0.281 N/mm^2^ for the long-axis and 0.101–0.179 N/mm^2^ for the diagonal left ventricular samples. In Table 2 the median and IQR of the elastic moduli for all tissues and all ranges are listed and in Figure 5 an exemplary plot of all average loading curves for all aorta samples is shown.

In Figure 5 all average loading curves by tissue are plotted. In summary, three different material behaviours were observed: quasilinear with just a slight curvature for the vessels (aorta and pulmonary artery), long toe region followed by an exponential increase in stiffness (strain hardening) for the right ventricle and the left ventricle short-axis and an immediate exponential increase in stiffness, followed by an early rupture for the left ventricular long-axis and diagonal samples.

### 3.2 Silicones

With certain silicones and silicone mixtures, there was a minor decrease in stiffness from the first 20% of deformation, compared to the stiffness between 20% and 40% deformation and then a slight increase for the deformation between 40% and 60% measurable. This can be seen in the median and IQR data of the elastic moduli for the silicones in Table 4. In general, the relation between the applied strain and the measured stress was mostly linear. Due to viscoelastic effects, the stiffer the silicone or silicone mixture was, the more difficulty it had with adapting to the unloading rate, thus leading to compression and initially negative stress values for very small strains, below 0.05.

**TABLE 4:** Elastic moduli of the silicones and silicone mixtures Median and IQR of the elastic moduli of the silicone and silicone mixtures. Lower and upper IQR are shown for all samples for each material and for 4 different deformation ranges.

		LIQR	Median	UIQR
Silicones	Range	E-Modulus [N/mm^2^]		
Dow Sylgard 184	0%–20%	1.407	1.487	1.506
	20%–40%	1.120	1.204	1.220
	40%–60%	1.301	1.411	1.486
	0%–60%	1.278	1.377	1.404
Wacker Elastosil RT601	0%–20%	1.388	1.409	1.425
	20%–40%	1.177	1.187	1.206
	40%–60%	1.313	1.321	1.353
	0%–60%	1.293	1.301	1.326
Elastosil Vario 15	0%–20%	0.345	0.346	0.350
	20%–40%	0.217	0.218	0.220
	40%–60%	0.183	0.183	0.185
	0%–60%	0.248	0.249	0.251
Dragon Skin 30	0%–20%	0.777	0.800	0.805
	20%–40%	0.563	0.572	0.577
	40%–60%	0.663	0.679	0.682
	0%–60%	0.669	0.682	0.688
Dragon Skin 20	0%–20%	0.478	0.482	0.484
	20%–40%	0.344	0.346	0.348
	40%–60%	0.401	0.404	0.405
	0%–60%	0.409	0.410	0.412
Dragon Skin 10S	0%–20%	0.315	0.316	0.317
	20%–40%	0.212	0.213	0.213
	40%–60%	0.200	0.201	0.202
	0%–60%	0.242	0.243	0.244
Ecoflex 00–30	0%–20%	0.064	0.065	0.065
	20%–40%	0.048	0.048	0.048
	40%–60%	0.040	0.040	0.041
	0%–60%	0.051	0.051	0.051
Ecoflex 00–20	0%–20%	0.049	0.050	0.051
	20%–40%	0.038	0.038	0.039
	40%–60%	0.033	0.033	0.034
	0%–60%	0.040	0.041	0.041
Ecoflex 00–10	0%–20%	0.028	0.028	0.029
	20%–40%	0.019	0.020	0.020
	40%–60%	0.018	0.019	0.020
	0%–60%	0.022	0.022	0.023
Dragon Skin 30 - Ecoflex 00–10 50/50	0%–20%	0.120	0.121	0.121
	20%–40%	0.088	0.089	0.089
	40%–60%	0.098	0.100	0.101
	0%–60%	0.102	0.103	0.104
Dragon Skin 20 - Ecoflex 00–30 50/50	0%–20%	0.215	0.217	0.219
	20%–40%	0.156	0.157	0.159
	40%–60%	0.160	0.163	0.165
	0%–60%	0.177	0.179	0.181
Dragon Skin 10S - Ecoflex 00–30 80/20	0%–20%	0.229	0.230	0.231
	20%–40%	0.157	0.157	0.158
	40%–60%	0.147	0.147	0.149
	0%–60%	0.177	0.178	0.179
Dragon Skin 10S - Ecoflex 00–30 60/40	0%–20%	0.190	0.191	0.192
	20%–40%	0.132	0.133	0.133
	40%–60%	0.122	0.122	0.124
	0%–60%	0.148	0.149	0.150
Dragon Skin 10S - Ecoflex 00–30 40/60	0%–20%	0.145	0.145	0.146
	20%–40%	0.102	0.102	0.103
	40%–60%	0.091	0.091	0.092
	0%–60%	0.112	0.113	0.114
Dragon Skin 10S—Ecoflex 00–30 20/80	0%–20%	0.097	0.098	0.098
	20%–40%	0.069	0.070	0.070
	40%–60%	0.059	0.059	0.060
	0%–60%	0.075	0.076	0.076

**FIGURE 7:**
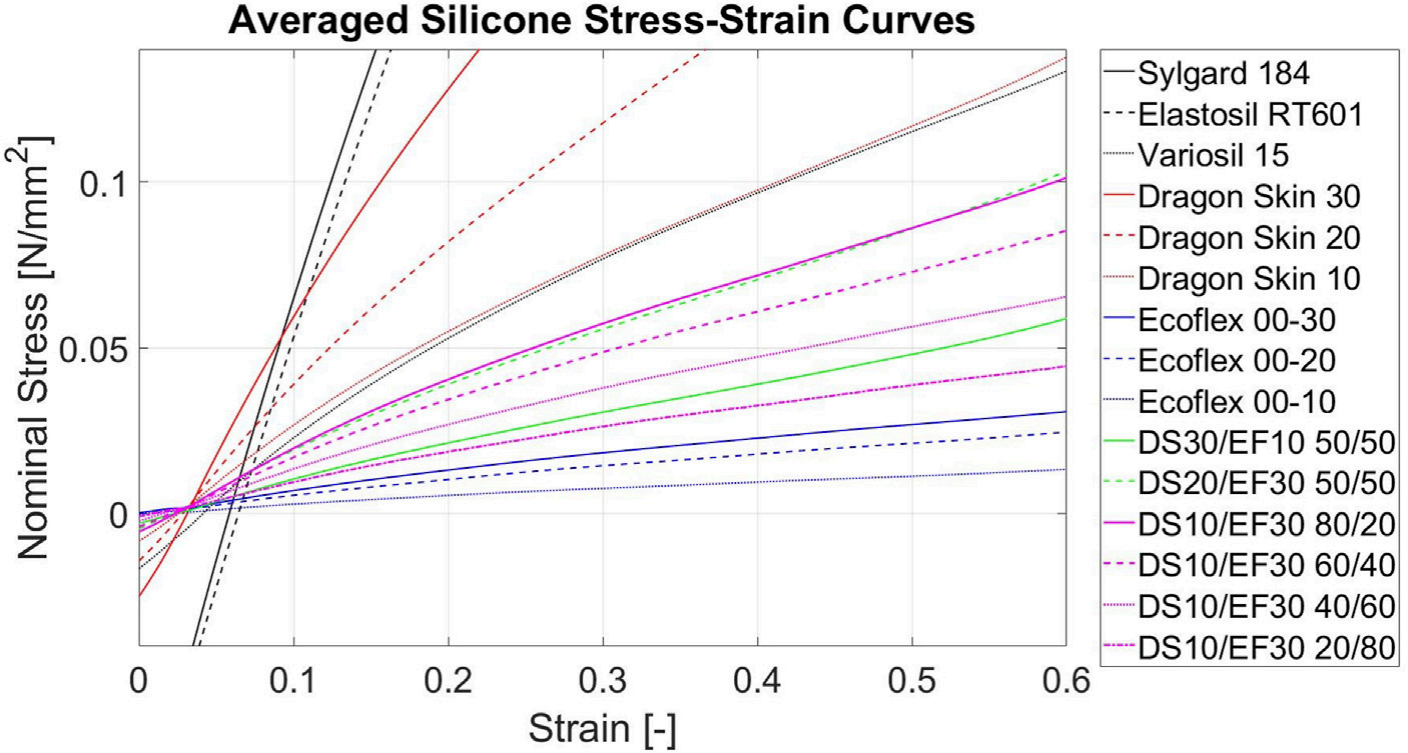
Stress-Strain curves of the silicones and silicone mixtures Averaged curves of the 15 silicones and silicone mixtures. All curves are averaged over 10 samples and the last 7 loading cycles. DS = Dragon Skin, EF = Ecoflex.

The two most commonly used silicones for cardiovascular phantoms (Sylgard 184 and Elastosil RT601) showed an almost linear stress-strain behaviour with nearly parallel loading and unloading curves that sometimes even crossed over (Figure 7). The Elastosil Vario 15 on the other hand, showed a strain softening behaviour in the first 20% of deformation followed by a linear curve. Thus, the elastic modulus decreased while loading. The pure Smooth-On silicones (Dragon Skin 30/20/10S and Ecoflex 00–30/20/10) had almost congruent loading and unloading curves and nearly linear behaviour. The Ecoflex only showed a slight stiffness reduction at the beginning, followed by linear behaviour. The moduli of elasticity corresponded with the silicones Shore values (Table 1; Table 4). The tested silicone mixtures behaved similar to the Ecoflex samples with strain softening until 20% deformation followed by a linear progression. The moduli of elasticity always laid in-between the two pure parental compounds (Table 4; Figure 7). A summary of the average silicone plots is shown in Figure 7. The Sylgard 184 and Elastosil RT601 samples were much stiffer than the others. The Elastosil Vario 15 was almost identical to the Dragon Skin 10Slow. While unloading, in contrary to the porcine cardiovascular tissue samples, the pure silicones all went into compression, showing negative stress values before reaching their initial length at 0 strain.

### 3.3 Additive manufacturing materials

**FIGURE 8:**
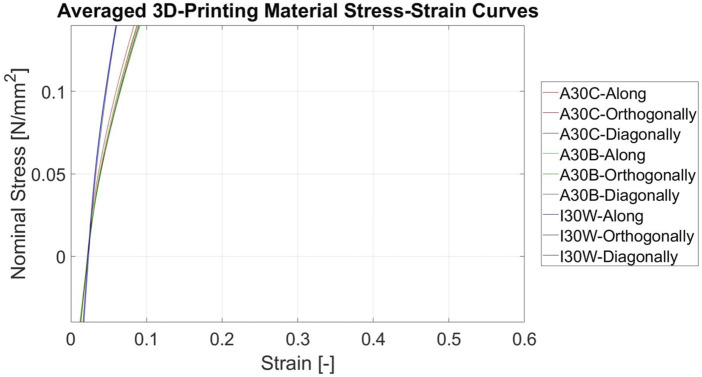
Stress-Strain curves of the 3D-printing phantom materials Curves of the three 3D-printing phantom materials (Agilus30 Clear (A30C), Agilus30Black (A30B) and IORA Flex 30A White (I30 W)) in three different printing orientations with respective to the print head axis. Averaged over five samples and the last 7 loading cycles.

All 3D-printing materials showed a strong strain softening behaviour at the beginning, until 10%–20% deformation, followed by an almost linear behaviour for the rest of the deformation. The difference between the loading and unloading curves were larger, compared to the porcine tissue and silicones. Irrespective of color or printing orientation, the curves of all Agilus30 samples exhibited identical behaviour. The IORA Model Flex 30 White behaved similarly in the above-mentioned points as the Agilus30 materials, while being overall stiffer (Figure 8; Table 5). Similar to the pure silicone samples, all 3D-printing samples displayed negative stress values (compression). The overall magnitude of compression was higher than for the silicone samples.

**TABLE 5:** Elastic moduli of the 3D-printing phantom materials Elastic moduli of the 3D-printing materials median, lower IQR and upper IQR over all samples for each material and their printing orientations and for 4 different deformation ranges.

		LIQR	Median	UIQR
Material	Range	E-Modulus [N/mm^2^]		
Agilus30Clear Along	0%–20%	1.879	1.879	1.588
	20%–40%	0.693	0.693	0.667
	40%–60%	0.556	0.556	0.554
	0%–60%	1.042	1.044	0.940
Agilus30Clear Orthogonally	0%–20%	1.718	1.839	1.689
	20%–40%	0.673	0.684	0.669
	40%–60%	0.549	0.550	0.547
	0%–60%	0.980	1.024	0.968
Agilus30Clear Diagonally	0%–20%	1.754	1.824	1.677
	20%–40%	0.683	0.689	0.679
	40%–60%	0.551	0.555	0.549
	0%–60%	0.997	1.026	0.971
Agilus30Black Along	0%–20%	1.813	1.827	1.796
	20%–40%	0.729	0.744	0.723
	40%–60%	0.611	0.625	0.606
	0%–60%	1.051	1.067	1.042
Agilus30Black Orthogonally	0%–20%	1.748	1.762	1.705
	20%–40%	0.727	0.731	0.725
	40%–60%	0.618	0.618	0.609
	0%–60%	1.034	1.037	1.016
Agilus30Black Diagonally	0%–20%	1.780	1.793	1.767
	20%–40%	0.735	0.737	0.731
	40%–60%	0.616	0.618	0.615
	0%–60%	1.044	1.050	1.038
IORA Flex 30A White Along	0%–20%	2.724	2.842	2.456
	20%–40%	1.179	1.183	1.160
	40%–60%	1.113	1.143	1.097
	0%–60%	1.682	1.713	1.586
IORA Flex 30A White Orthogonally	0%–20%	2.668	2.735	2.617
	20%–40%	1.184	1.185	1.176
	40%–60%	1.140	1.141	1.132
	0%–60%	1.670	1.692	1.645
IORA Flex 30A White Diagonally	0%–20%	2.779	2.827	2.692
	20%–40%	1.186	1.197	1.184
	40%–60%	1.133	1.142	1.111
	0%–60%	1.692	1.722	1.670

## 4 Discussion

**FIGURE 9:**
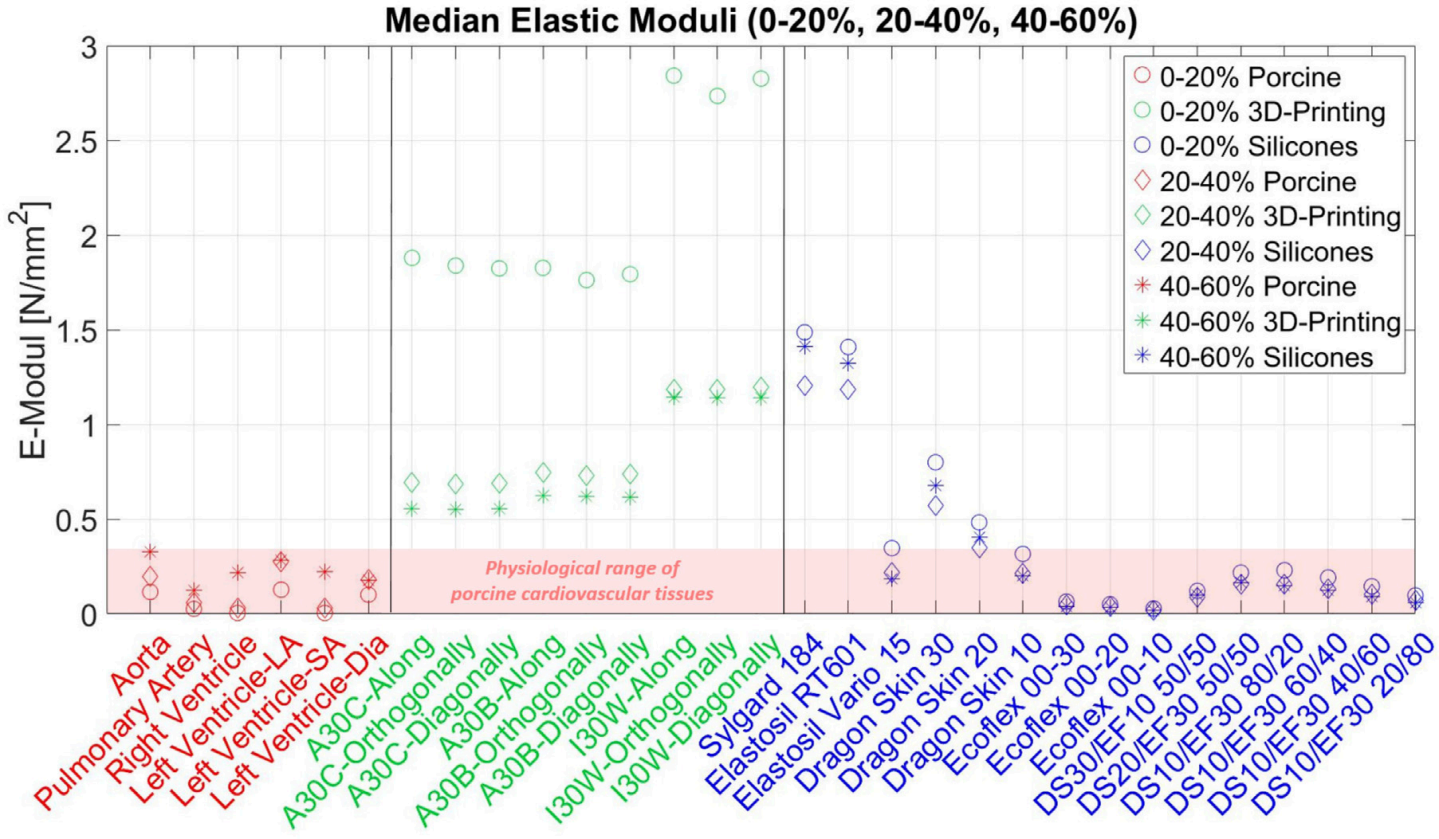
Median Elastic-Moduli among all materials for 3 strain ranges Median of the elastic moduli of all tested phantom materials for the 3 different strain ranges (0–0.2, 0.2–0.4 and 0.4–0.6 strain). The color identifies the material type and the marker shows the corresponding strain range.

**FIGURE 10:**
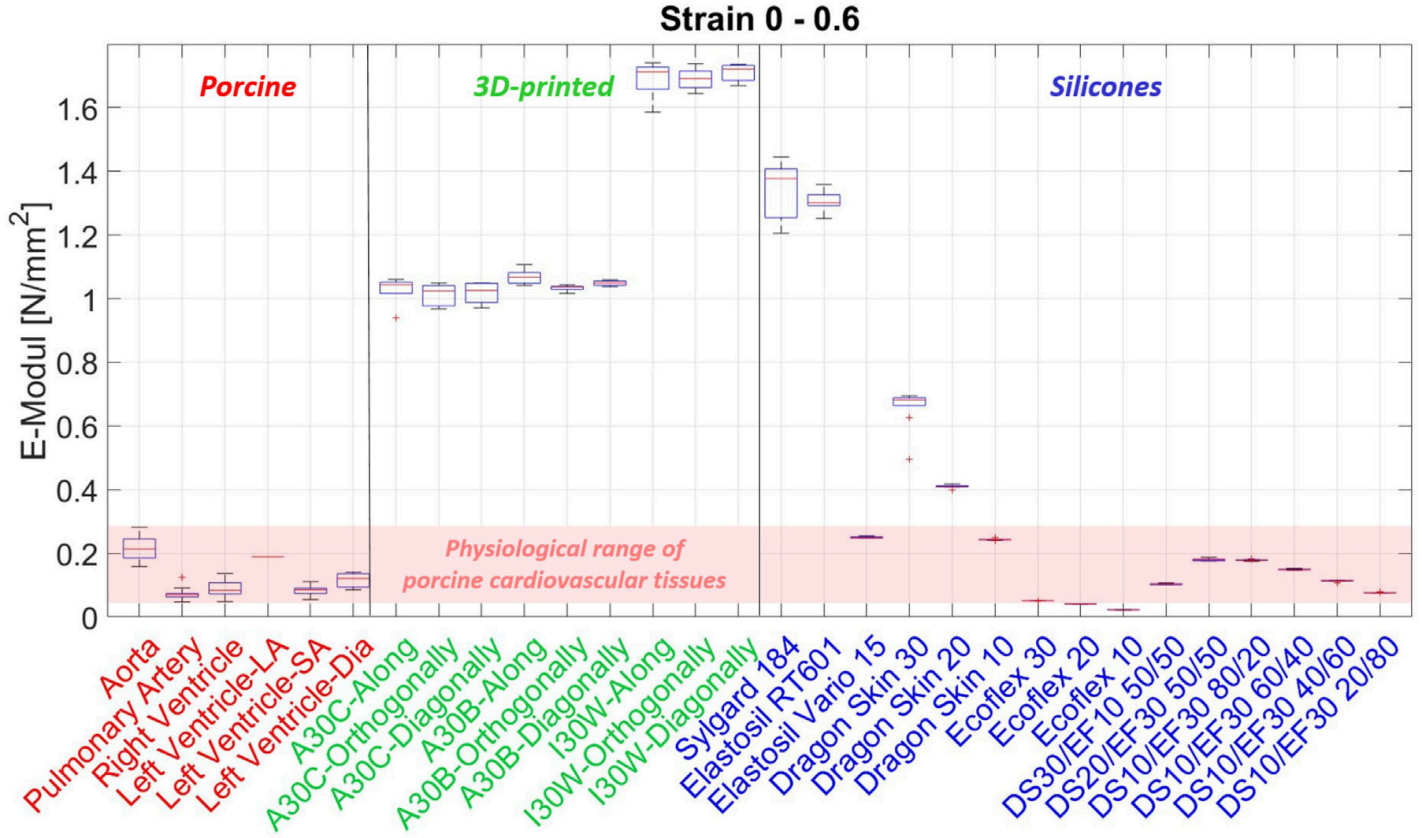
Boxplots of all materials results over the whole strain range Boxplots of the elastic moduli of all tested materials for the total strain range of 0–0.6 strain. The black vertical lines separate the different material types (porcine cardiovascular tissues, 3D-printed phantom materials and silicone phantom materials).

In summary, in our study we could show that currently used silicones and 3D-printing materials are not compliant/soft enough to mimic cardiovascular tissues (Figures 9, 10). Additionally the stress-strain response of the artificial materials was different and tending more to softening at the beginning of deformation, compared to the late strain hardening of soft tissues, due to fiber recruitment.

The similar behavior of the vascular tissue samples (aorta and pulmonary artery) can be explained due to their similar collagen composition and muscle fiber type. In contrary, the myocardial tissues samples show a different behavior due to a different composition and muscle fiber type. The similarity of the left ventricular short-axis to the right ventricular samples shows that the general buildup of the myocardium is similar and that the difference in stiffness arises not only, but mainly from the different wall thicknesses and fiber orientation (Nemavhola, 2021/04; Liu et al., 2022). The latter is also supported by the fact that all left ventricular long-axis and diagonal samples ruptured orthogonally or nearly orthogonally to the deformation axis in the gage length area and at a similar extent of deformation, whilst the other two myocardial samples stayed intact. This can be explained on one hand by the different physiological strain ranges, which are much higher for vascular tissue than for myocardium (Voigt and Cvijic, 2019). The expected strain-hardening behavior is most probably attributed to the staged fiber recruitment of soft tissues (Zhalmuratova et al., 2019).

For the most commonly used cardiovascular phantom materials (Agilus30, Elastosil RT 601, Sylgard 184), it can be said that Agilus30 was the closest to biological elasticity properties but still far too stiff. However, the pronounced hysteresis and resulting compression regime when unloading Agilus30 significantly differs from the biological tissue behavior. Thus the material needs to be optimized to become softer.

The measured Smooth-On silicone mixtures of Dragon Skin and Ecoflex showed elastic moduli similar to the porcine tissues, while the difference in curvature was minimal. Also, they showed little to no compression when unloading, similar to the porcine tissues. The next step would be to create individual mixtures for each cardiovascular tissue to create materials that are even closer to physiological behavior. Therefore, future implementation of mimicking fiber recruitment in 3-printed and silicon phantoms could potentially overcome this issue. The outcomes obtained for the silicones and their combinations aligned with the anticipated values based on the advertised Shore hardness by manufacturers.

Furthermore, the measured values of the mixtures consistently fell within the range of the two distinct parental compounds. The occurrence of compression during unloading signifies that the imposed relaxation rate, despite being lower than the deformation rate, exceeded the capability of the silicones to accommodate the deformation and also shows the viscoelastic nature of those materials. As the used deformation and relaxation rate corresponds to a heart rate of 10 beats per minute for the deformation of 60%, the effect would be much stronger for physiological heart rates of 60 beats per minute. The same is true for 3D-printing materials, but they also demonstrated significantly larger hysteresis than the silicones, which were closer to the porcine tissues. A significant advantage is the printing orientation’s independence, which can only be regulated for an entire phantom at a global level. To clarify scientifically, the printing orientation’s independence refers to the ability to print a model without considering the direction of printing (orientation of the phantom model in the build volume of the printer) during the planning of the print.

### 4.1 Outlook

Based on our results, the testing protocol could be extended to additional deformation and relaxation rates to account for strain rate-dependent behavior and viscoelastic effects and multiple peak deformations for the myocardial samples’ strength and ruptures. Applying more realistic testing conditions (temperature, humidity, and general cell viability) would lead to a more realistic depiction of the tissue properties. Concerning the fiber-based anisotropic material behavior, AM-materials look more promising, as with voxel-printing (Illi et al., 2022), a microstructure could be built in the core of the sample or phantom to mimic the fiber recruitment and also to create softer, more compliant materials. Furthermore, in the case of whole heart phantoms, AM would also make it much easier to combine different materials for the different tissues in a single model. These two points are very challenging to achieve with silicone casting. Such voxel-printing samples with different elasticity and orientation have already been tested with a Shore A durometer and shown to be at around 5–10, putting them in the range of cardiovascular tissues measured in this paper. In-depth uniaxial tension tests of those materials with different elasticities, fiber orientations, and content will follow.

### 4.2 Limitations

The primary limitation is that we tested dead porcine cardiovascular tissue and not living human tissue (Martin et al., 2011). We can assume that the difference between porcine and human tissues is smaller than the measured difference between the currently used artificial phantom materials and the porcine tissue (Ferrara et al., 2016).

Moreover, the mechanical behavior of cardiovascular tissues is profoundly influenced by their dynamic nature, and their inherent mechanical activity. Therefore, uniaxial deformation tests utilizing a solitary peak strain and strain rate fall short of encompassing the full mechanical response spectrum of these tissues. Consequently, no artificial, non-tissue engineered material can comprehensively replicate their behavior under all states and conditions. Nevertheless, for an initial evaluation of the situation, we believe that our selected parameters align with physiological conditions.

As soft tissues have anisotropic material behavior due to the fiber orientation and a biaxial response, a biaxial tension-testing system with a biaxial strain measurement or at least a biaxial strain measurement would render a more accurate depiction of the mechanical properties. We focused on the isotropic bulk properties, as anisotropic material behavior will be even more challenging to reproduce in phantoms.

As measurement for the sample deformation we used the jaw displacement of the setup, this is not absolutely true, as those two can differ. But as our setup was built to withstand up to 10 kN and the largest force we saw during our tests did not exceed 90 N the error is negligible. Nevertheless, we did a trial to proof our assumption with a video-extensometer and an AM sample with printed markers.

Due to the generation process of the vessel samples the residual circumferential and logitudinal stresses are released during the dissection process, before the generation of the sample geometry. Thus, the sample geometry was not distorted by this release. Nevertheless a bias in the mechanical response, due to the lack of those residual stresses could not be avoided.

The fiber orientation in the myocardium changes from location to location and sample orientation, but also from epi-to endocardium. Thus an additional orientation in the transmural direction would be needed, but due to limit in myocardial thickness, creation and testing of such a sample would be very difficult without specific equipment. With the filleting of the left ventricular samples, the endocardium was cut away, and thus our measurement results only represent the mechanical properties of the outer 10 mm of the myocardium and not the whole thickness, which can lead to bias. When comparing the filleted to the non-filleted data, there was no indication of a relevant bias. However, this would need to be investigated thoroughly in case of future biaxial strain measurements. Furthermore, due to the ruptures of the left ventricle long-axis and diagonal samples, only parts of the first loading cycle could be analysed, thus leading to overestimating those due to the adaptation effect. As the measurements were taken at a laboratory room temperature of 21°–23°C and the samples were prevented from drying out by covering them with a paper towel drenched in a phosphate-buffered-saline solution, a difference to living tissue inside a body has to be expected (Chow and Zhang, 2011).

### 4.3 Conclusion

We could demonstrate that the moduli of elasticity of our softer silicone mixtures are more in agreement with porcine cardiovascular tissues than 3D-printed materials. No artificial material is currently suitable to reproduce physiological tissue properties over the physiological range of deformation. While Agilus30 seems to still be the best material for direct AM of cardiovascular phantoms, Smooth-On silicone mixtures of Dragon Skin 10Slow and Ecoflex 00–30 proved to be an ideal material for indirect AM casting. The potential enhancement of the physiological mechanical behaviour of future 3D-printed phantoms may lie in the implementation of mimicking fibre recruitment while simultaneously lowering the general stiffness.

## Data Availability

The raw data supporting the conclusion of this article will be made available by the authors, without undue reservation.

## Abbreviations

AM, additive manufacturing; 3DPSP, 3-dimensional patient-specific phantom.
